# Teaching psychiatry through movies: the PTSD in *Brothers*: film analysis

**DOI:** 10.3389/fpsyt.2026.1841640

**Published:** 2026-06-11

**Authors:** Jasper Crokaert, Yasmine Bouayed, Clara Daher, Gerard Calzada

**Affiliations:** 1 Department of Psychiatry and Mental Health, Geneva University Hospitals, Thônex, Switzerland; 2 Faculty of Medicine, University of Geneva (UNIGE), Geneva, Switzerland; 3 Private practice, Geneva, Switzerland

**Keywords:** cinema, education, family system, health care system, post-traumatic stress disorder, PTSD, public health, semiology

## Abstract

**Background and context:**

This article originates from the work of students who participated in the “Psychiatry in Cinema” course, offered annually to medical students at the University of Geneva. An introduction to psychiatry is carried out through the visual support offered by cinematographic works, which have the advantage of depicting psychiatric symptoms in a concrete way to facilitate the teaching of semiology by giving substance and materiality to sometimes abstract theoretical concepts, while also addressing certain stigmas and stereotypes linked to the mental illnesses portrayed in these films, even before students begin their clinical rotations. This article presents their work and reflects their ongoing learning by highlighting the identification of symptoms in the film and matching them with the criteria defined in theoretical reference manuals. *Brothers* is an American film that tells the story of a soldier named Sam, who returns from the front and struggles to readjust to family life. As a result of the horrors he witnessed, he develops posttraumatic stress disorder.

**Objective:**

This article discusses posttraumatic stress disorder as depicted in this cinematic work. The different clinical signs that contribute to the diagnosis will be discussed. Secondly, a critique of the staging and the manner in which the protagonist’s internal experience is portrayed will be presented. By reading this article, the reader will understand whether the representation of this disorder is consistent with psychiatric diagnostic manuals, as well as with the subjective experience of patients. Thirdly, the social representation of the disorder proposed by this film will be addressed. Does this film offer a stigmatizing image of PTSD, and how might this influence the public’s perceptions of patients suffering from this disorder? Finally, the representation of psychiatric care will be addressed; indeed, this point is particularly interesting in the context of American soldiers returning from combat profoundly affected by their traumatic experiences of war.

**Results and conclusion:**

This film raises an important political question regarding the therapeutic care of soldiers who have not always been treated in an appropriate, understanding, and integrated manner upon their return. The methods used by the director to highlight this issue will be discussed and commented on extensively at the end of this article.

## Highlights

• The movie depicts an American veteran affected by posttraumatic stress disorder and shows how this disorder affects his relatives and family system upon his return.
• This article proposes linking the concrete cinematographic representation of this disorder with theoretical concepts presented in recognized manuals, with the aim of introducing psychiatry and mental health.
• The stigma associated with the disorder, as conveyed by the movie, as well as the representation of violence by affected patients, will be addressed and discussed, together with any supposed political messages carried by the movie.

## 1 Introduction: Context and aim of the article

Movies are entertaining but are also useful in psychiatric education (1), social change, and offer a diverse and rich teaching tool complementary to more traditional and classical clinical settings (2). Psychiatrists cannot ignore that movies are tools of mass communication and often convey positive or negative conceptions about mental health, leading to the establishment of frameworks for understanding such movies or even mutual exchanges that contribute to the creation of didactic tools (1, 3–18). For example, in teaching psychiatry through movies, psychopathology is illustrated, while understanding and empathy are fostered in medical students by preparing them to assess patients (2, 19). Furthermore, stigma and misconceptions are addressed among individuals ranging from high school students to medical professionals, thereby opening a debate that challenges prejudices in everyday life and in clinical settings (1). Movies also encourage debate and critical analysis of clinical cases, the doctor–patient relationship, and the accuracy of diagnoses, thereby enhancing the critical thinking of students and future doctors (14). Teaching psychiatry through movies is feasible, implementable, and useful for the recognition of mental disorders, although learning ability and long-term effects should be studied in future research (2). Moreover, as a recent systematic review suggests, research in this area should be standardized, methodologically more rigorous, and more focused on the effectiveness of learning outcomes (16).

Our article proposes a phenomenological analysis of the movie *Brothers*, mainly from psychopathological and psychotherapeutic perspectives in an educational setting, aimed at enhancing critical thinking, while also addressing the burden of mental health on relatives, as well as social representations and stigma. Finally, our article addresses the possibility of hidden political messages related to foreign policy and inadequate mental healthcare and support for returning veterans, their families, and relatives in a postwar context. This movie could lead to heightened awareness of the psychological consequences of war, its human cost, and the gaps in support and treatment systems that need to be addressed.

## 2 Plot of the film

US Navy captain Sam Cahill, married to his high school sweetheart Grace and father of two little girls, is about to return to Afghanistan on a mission. His younger brother, Tommy, is fresh out of prison. During a family meal, the viewer discovers that the family situation is marked by tension and internal friction. The father of the two men, Hank, is a former Marine who served in the Vietnam War. He does not conceal his preference for his eldest son, Sam, nor his disappointment in his youngest son, Tommy. Initial tension is also evident between Tommy and Grace.

Deployed in Afghanistan, Sam and his team are involved in a helicopter crash, from which Sam and Joe Willis (a private) are the only survivors. The duo is imprisoned by Taliban warriors, who subject them to both physical and psychological torture. Under duress, Sam is forced to beat his brother-in-arms to death or face being killed himself. The idea of reuniting with one’s family appears to prevail and precipitate, if not facilitate, the act of violence.

On the other side of the globe, the members of Sam’s family believe him to be dead and strive to move forward despite the tragedy through a difficult psychological process of mourning, seeking a new family balance in Sam’s absence. Tommy, initially presented as irresponsible, gradually takes on the role of his older brother by taking care of his two nieces, Isabelle and Maggie. A rapprochement is also observed between Tommy and Grace, who are initially reluctant to like each other, with its culmination being a kiss shared by the fire during a moment of intimacy, as Tommy has lost his brother while Grace lost her husband.

During a raid on the Afghan base, Sam is found by the US military before being repatriated. When his family hears the news, the viewer observes mixed and ambiguous feelings among his family members, namely a mixture of joy and bewilderment. Grace, Tommy, Isabelle, and Maggie had found a new balance in Sam’s absence; a new homeostasis of the recomposed family unit, which his unexpected return disrupts. For his part, Sam struggles to reintegrate into his own family and appears haunted by memories of the past that he cannot seem to share with his loved ones. The relationship with his daughters gradually deteriorates, and the daughters seem to miss their uncle Tommy, who has withdrawn since his return. In addition, through various events and exchanges, Sam realizes that he is only a shadow of what he used to be.

The film ends with a psychic decompensation on Sam’s side, as he threatens to commit suicide or harm his relatives. At the urging of his brother and his wife, he is admitted to a veteran’s psychiatric hospital for treatment.

## 3 Film analysis and discussion

The film begins symbolically with the display of an American flag, already announcing that the plot of the film is supported by patriotic elements and that the USA plays a central role in the film. The movie itself was addressed to the country, and the protagonists come to represent the country more broadly.

The contrast between the two brothers is striking, with the eldest son represented as “responsible” and “exemplary” due to his service to his country, according to the father. In contrast, the younger brother is presented as a drinker, irresponsible, and somewhat of an outcast (Grace pays for his drinking, he borrows his deceased brother’s car, and he drinks from the bottle). Sam, in a way, identifies with his authoritative, responsible, patriotic, and veteran father, while his younger brother does not and instead occupies a different role. The father’s speech, with its rigid and inflexible tone, can symbolically represent the Superego of the USA in a metaphorical sense, insofar as it was commonly accepted, and even expected, during this period that serving one’s country by going to war was honorable, as Hank did, and as Sam also did, whereas this discourse is experienced in a more guilt-ridden way by his brother Jimmy, who remains in the country. The act of going to war can be seen as an injunction, a demand, a duty, and an order, amounting to Sam and Jimmy’s father unconsciously setting up a rivalry between the two brothers. It is also interesting to note that the father is presented as polished, composed, rigid, somewhat cold and distant, and even unattainable.

It is also interesting to note that family meals catalyze and facilitate exchanges, structuring the scenes with a certain rhythmicity by bringing the different protagonists closer together at key moments in the film, namely before Sam’s departure as well as upon his return.

A parallel can be drawn between Jimmy’s arrival and Sam’s departure (at the center of the plot—the film being titled *Brothers*), which occur almost simultaneously and, in a way, immediately establish the first milestones of the film’s plot. Sam’s difficulty in removing his ring to place it around his wife’s neck by means of a necklace immediately signals that the relationship between Sam and Grace is at the center of the film.

When Sam is absent, when the elder brother no longer fulfills his role or paternal function (4–15), Jimmy positions himself in the vacuum left by Sam, “replacing” him in a natural way and finally beginning to exhibit more responsible behavior by identifying (5), possibly with his absent brother on the one hand but eventually also with his father on the other, as the position become vacant and sibling rivalry has been removed.

On the other hand, through family interactions and tensions, it appears that the father of the family, a Vietnam veteran, was also affected by his experience of war and that, by keeping his experience to himself without ever addressing it, this led to abusive alcohol consumption as well as intrafamily violence against his wife and children, in a movement of transgenerational repetition of the same (6).

The staging of the kitchen and the renovations that take place there also seem to play a central role in the unfolding of the film. The old kitchen is renovated by Jimmy and his associates before the contrast is quickly highlighted with the torture to which Sam is subjected in Afghanistan, where the viewer is noticeably positioned as experiencing injustice and guilt in relation to Jimmy’s friends, who drink beer and wear Sam’s clothes (symbolically taking his place), while Sam is subjected to unbearable torture. The new kitchen, fitted out by Jimmy, symbolizes the newly obtained family balance, where he finally assumes and occupies the role of responsible father instead of his brother, acting as a substitute for the paternal function in the family before eventually replacing him entirely (12); this includes not only taking his place with his wife but also with his children in moments of pleasure and everyday life. The kitchen becomes the epicenter of the home, where exchanges take place, thus representing the nucleus of the family unit. It should also be noted that Jimmy’s increasing assumption of responsibility plays a central role in the film, as he leaves behind his habits as a thug and thief, to the point of apologizing to the bank representative he had previously threatened during a robbery. The latter remarks in a symbolic and eloquent way, as a subliminal message whose meaning is revealed at the end of the movie, that she can “breathe again”.

For his part, captive and tortured, Sam is confronted with the choice of killing or being killed. The psychic pressure for Sam becomes unbearable, and he *reorganizes* himself “to survive” by *detaching himself from his emotional experience* through a psychic survival mechanism of emotional disconnection, well described and studied in the literature on the subject (7, 8, 13, 20, 21). It is also interesting to note that the perpetration of violence seems to be a criterion of severity and complexity for posttraumatic stress disorder (PTSD), probably through a feeling of shame and guilt, thereby questioning the notion of powerlessness that previously played a central role (14). When he returns, he is frozen and fixed in his past, without access to his emotional experience, making him less flexible in interactions and hindering his parenting abilities as well as the educational framework he provides his children. Upon Sam’s return, the new equilibrium of the recomposed family unit bothers and annoys him. The glasses are arranged and stored in the kitchen in an obsessive manner to *regain control* and externalize internal anxieties during a *passage from phantasm to action*. When his daughter taunts him and claims that Jimmy has also replaced him in Grace’s bed (which is not true), Sam’s frustration becomes unbearable, and he can no longer contain himself. He knocks down the new kitchen with the help of a bat, symbolically breaking the newly established balance and homeostasis formed in his absence. This act represents a movement of “displacement” from the anger he feels towards his brother Jimmy, who has replaced him in this new equilibrium. The new kitchen, as mentioned above, serves as the symbol of this shift, and his destructive action redirects the anger towards Jimmy himself.

Sam is irritable, staying away from the crowd when his family happily indulges in the pleasures of skating. He is also less malleable in his functioning and can no longer adjust to the needs of his children (he does not laugh at their jokes, misfits in their hugs; the girls preferred the atmosphere when Sam was away), to the point where his daughters begin to fear him and miss their former equilibrium and recomposed family in Sam’s absence. Sam is also less flexible and less affectionate in his relationship with his wife, as he rejects her displays of affection because he is busy shaving. It is also interesting to note that the physical marks of the torture suffered also “embody” and represent psychological pain and suffering. Sam later proves to be hypervigilant and seems to relive moments of constant danger in a context of mistrust when he walks around his home armed with his gun, worried about the noises of nearby dogs. At the dinner table, when his daughter refers to the turtle that hides under its shell when in danger, it appears to function as a subliminal message that director Sheridan is trying to convey by referring to the emotional *detachment as a defense mechanism* in the context of complex PTSD. Sam’s internal anxiety manifests in hypervigilance (22) in relation to his environment, resulting in a need for control as well as an intolerance to frustration (23) in the event of a loss of control (he can no longer tolerate the slightest noise—*misophonia*—nor the slightest unforeseen event), which in turn manifests as a significant internal tension expressed at the psychomotor level, namely in an obvious bodily agitation more accentuated in his hands and fingers.

Taking a step back and acknowledging that the two brothers represent the same father before and *after* the war, the allostatic burden of war trauma brought home affects not only the protagonist but also imposes a significant burden on his entire family and loved ones. With hindsight, while drawing the line, a possible political message can be discerned in the director’s critique of the lack of appropriate care for returnees from war (Afghanistan, as well as Vietnam and Iraq), emphasizing that soldiers are abandoned to their fate after committing themselves to the homeland out of patriotism and in response to societal and national pressure. As for care, it is first evoked in an elegant way when Diana, Jimmy’s last friend, who is studying nursing, innocently and subtly suggests that, although Marines are trained to fight war, “no one can be trained to kill”, because even Marines remain “human”.

This movie serves as a reminder of the human consequences of foreign policies, a critique of masculinism and duty as rigidly expected by the brothers’ father, and more widely a humanist critique of modern war and its hidden and overseen costs (10).

The question of care for mental illnesses and responsibility for this care is posed once again in a poignant way at the end of the cinematographic work, when the artistic work conveys a potentially political message by questioning the interventionist policy—the “War on Terror” of the USA at that time (Vietnam and Afghanistan)—while opening up a space for reflection on the care to be given to returnees and veterans of these two wars. This message is also conveyed in a subtle way: “Save our fallen soldiers”. As is often the case in psychiatry and psychotherapy, care involves putting words to emotional experience in order to free oneself from it: “I killed Joe Willis, can I live again?”, alluding to the banker who explains, much earlier in the film, that she is able to “breathe again”. Finally, the letter read at the end of the film reminds us of the beginning, closing the loop and ending the film on a more romantic note.

At last, relatively to the title of the movie *Brothers*, it could be hypothesized that Joe replaces Sam throughout his life: within the family by taking on responsibilities, with his children as a surrogate father, and with his wife, as if the two brothers represent the same individual. In fact, it could be that the two brothers represent two faces of the same coin, which symbolically emphasizes, by accentuating the delta representing the impact and the cost of war, what war did to the families of US veterans, while raising the question of how to rehabilitate and reintegrate those veterans into American society.

## 4 What you need to know

*Brothers* was directed by Jim Sheridan and released in 2009 (24). It is a readaptation of a Danish film of the same name released 5 years earlier. The cast includes Natalie Portman as Grace, Jake Gyllenhaal as Jimmy, and Tobey Maguire as Sam, contributing heavily to the wide distribution of the film, which was nominated in various categories at the Oscars without winning any. Tobey Maguire’s performance, however, contributed to his nomination in the Best Actor category at the 67th Golden Globe Awards.

The film could be classified into several categories of war films and be valued across multiple facets. First, the film could be considered within the “Sacrifice of the Fighter” type, whose heroic protagonist is deeply committed to his homeland in a patriotic impulse. Secondly, the film highlights the soldiers’ loss of innocence in contact with war and addresses the atrocities committed therein. Thirdly, the film would also subtly denounce the promilitarist and interventionist American system that sacrifices its men by sending them to war, while subtly criticizing the lack of adequate care upon their return.

From a reception perspective, reviews of the film are quite mixed. The film has a rating of 7.1/10 on IMDb. The acting is appreciated, but it is rather the direction that displeases audiences. In his *New York Times* article, Scott criticized the film’s lack of direction, saying it left “a feeling of a half-finished movie” (25). The sequence of events appears suboptimal: after a long exposition articulated through the development of the relationship between Grace and Tommy, their story ends abruptly with Sam’s return, without their romance being further elaborated. The director then concentrates on the detailed staging of Sam’s PTSD, but again in a rather external way, without providing access to the inner workings of his psychic life.

The context of the film’s release is noteworthy in that public awareness was rising in the postwar period of 2009 (2001 and 2003 interventions in Afghanistan and Iraq) concerning PTSD and growing suicide rates in veterans returning from protracted conflicts. The film appears to mirror real-world anxieties and apprehensions about the inadequacies of support and treatment for these veterans. In fact, the long waiting times for intervention, as well as understaffed and underfinanced support programs, are implicitly critiqued, leading, with Obama taking office in 2009 (the year of the film’s release), to reforms aimed at addressing these slow, bureaucratic, and under resourced healthcare systems. The reforms aim to address the healthcare gap in a holistic way, providing care for mental health and physical health, while also proposing family support (26) to help veterans reintegrate into civilian life and to help them find meaning to continue and move forward (11). To do so, rehabilitation programs should include screening, early intervention, mobile counseling, and the management of comorbid addictions (1, 3–17, 19–33). Furthermore, educational programs and access to resources should be enhanced to manage these *invisible wounds* (32) that war left on veterans, as is implicitly suggested by the film.

## 5 Phenomenological psychopathology

Sam is prone to dissociative complex (PTSD) (16, 17, 22, 26, 29) upon his return from the war in Afghanistan. Multiple traits are elegantly depicted, such as a gloomy gaze into the void, conveying a sense of constant absence in his eyes, as if he were detached from his loved ones as well as from his own internal experience. In addition, subject to a significant narcissistic collapse, Sam is emptied of desire as well as life momentum, hampered by this traumatic intrusion, which becomes the reorganizing epicenter of his psychic life.

To retain the diagnosis of PTSD, several criteria must be evaluated according to the ICD-11 (12) reference manual:

The first criterion to consider is exposure to one or more traumatic events. The patient must have witnessed a death-related event, serious injury, or threat of death, and there must be an element of fear, helplessness, or horror; Sam ticks all the boxes.

The second criterion, that of intrusive symptoms, is not clearly represented. Nevertheless, the viewer can assume their presence when Sam is absent, presenting a blank and lifeless stare despite the presence of his family. Even if not clearly stated, this blank stare could be due to *flashbacks* during these moments. The director could have included these elements in the form of *flashbacks*, auditory or visual hallucinations, revivals, reminiscences, or repetitive memories, dreams, or nightmares. Finally, a feeling of psychological distress at the mention of the traumatic event seems evident, as well as the physiological reaction that accompanies it when exposed to cues evoking this event.

The third criterion, concerning symptoms of avoidance of conditioned cues and blunting of affect, is also present. Sam is unable to discuss the traumatic events with his wife, who proposes to listen and support him. A restriction of affect, nonmodulated, leading to detachment from loved ones, is clearly evident. The viewer-spectator can observe this by comparing his behavior and attitude *before* and *after* the traumatic event to better perceive the difference or quantitative *delta*. Prior to his military deployment, the protagonist is depicted as a loving husband and father who interacts with his family in an appropriate manner while maintaining a satisfying and mutually supportive relationship with his brother. Returning from Afghanistan, Sam carries an allostatic load of unprocessed trauma, which surreptitiously intrudes into his multiple interactions with his disrupted family. Sam avoids his wife, struggles to reconnect with his two daughters, and has difficulty adjusting to them. He is also angry at his brother in the context of a suspected relationship between his brother and his wife. Scenes show Sam engaging in various household chores to keep himself busy, such as fixing his car or rearranging all the dishes in the kitchen, reflecting an unfulfilled search for meaning. These elements give rise to a feeling of a “clogged” and blurred future, in which the protagonist does not know how to reclaim his place in the new family dynamic that developed during his absence. These elements lead Sam to want to return to Afghanistan, as he begs his superior to send him back to the front. Paradoxically, this aspect goes against the criterion of stimulus avoidance and represents a less plausible element in the depiction of PTSD in the cinematographic work. By adding this element, the director may have intended to convey the discrepancy Sam experiences upon returning home, as well as the lack of meaning in his life, even in the presence of those he loves. For him, what makes sense is to return to fighting with his men in order to feel useful.

On the neurovegetative side, internal and permanent anxiety leads to a mimo-gesto-postural attitude of hypervigilance in Sam, manifested through incessant scanning and numerous attempts to control his environment and himself, such as when he obsessively puts away the glasses in the kitchen, from which irritability emerges in the event of loss of control. Sam is sensitive and irritable to certain neutral, even innocuous stimuli, such as various sounds and unforeseen events, going as far as episodes of anger, with a *clastic crisis* of great vehemence as its peak during the girl’s birthday meal. Finally, startle responses, anxiety, and hypervigilance leave no room for rest and tranquility necessary for sleep.

The criterion concerning the significant and functional impact of his symptoms on the patient’s daily life is also met. Sam’s social functioning, showing a regression to a previous level of functioning, is impaired and disrupted. The traumatic burden that Sam brings back to his family hampers the overall functioning of the family system when he interacts with its various members. The realization of this difficult and tense return adds secondary suffering for Sam, who, in an avoidance movement regarding family difficulties in which he does not find his place, combined with a desire to *flee forward* or return to a previous equilibrium, seeks to regain a place he considers his own by requesting to return to the front.

Finally, the question of a possible characterized and comorbid depressive episode arises in view of the clinical presentation; depression is comorbid with PTSD in approximately half of cases (8, 9, 29).

In conclusion, the criteria for complex PTSD are mostly correctly represented in the film. *Brothers* raises awareness of posttraumatic stress disorder while remaining relatively superficial, mainly by focusing on external representations rather than the internal experience of the condition. In fact, the disorder is approached in a manner consistent with diagnostic criteria, even though the *subjective experience* of the patient is not addressed with greater breadth and depth.

## 6 Social representations

Sam is initially portrayed in an idealized way as the American soldier within his family system, as a son, brother, father, husband, and model citizen committed to his country. Sam then evolves in a military setting, remaining patriotic to the end despite the physical and mental damage he endures. He is represented as having a strong capacity for control and composure throughout this period, until the fateful and pivotal moment when he kills Private Joe under pressure, driven by the desire to *return to his family*. A cold gaze gives the impression of a dead tree without leaves or life, a shadow of himself, marked by emotional detachment that fixes and freezes Sam in his functioning.

The stereotypes that emerge to illustrate PTSD manifest as soon as Sam returns. Sheridan portrays a man who is profoundly changed and unable to reintegrate into his family or find his place within a newly reconstituted system.

The criteria the director used to characterize PTSD, while accurately represented, function as vectors of certain *stigmas*. Representations of violence, impulsiveness, and even dangerousness are conveyed by this film, although this is not necessarily inherent to this psychopathological category, even if it has been observed among war returnees (30) and, more broadly, in relation to mental illness. Spectators, conditioned by the staging of this violence, may develop biased representations and fear regarding the supposed dangerousness of patients affected by the disorder in question, from which unjustified discrimination could result (31).

The use of these stereotypes also has positive facets, such as raising community awareness of the traumatic effects that war can have on soldiers, then on their families, and finally on American society in general. The lack of support and *follow-up* for veterans is highlighted in an acerbic and bitter manner at the end of the work. On the other hand, negative stereotypes are also present, namely the reduction of the disorder to a *cliché* or photo, depicting it in an external way without really providing access to Sam’s internal psychic experience.

In the end, very little space is left for the American healthcare system in the film. The film seems to allude to this in a subtle and subliminal way during the final denouement, as if Sheridan were advocating for the care of veterans and making a plea for possible *screening* upon their return, in order to set up appropriate follow-up tailored to their needs. The film may carry a political message in that it appears to point to the responsibilities of political institutions and groups in a context where, as with Sam’s father (himself a veteran who had never elaborated on his experience of Vietnam), American society at the time treated the suffering of ex-combatants as a social taboo. Transgenerational continuity (5) is elegantly emphasized through Sam’s identification with his father’s patriotic values. Indeed, Hank, Sam’s father, never gave meaning to his military experience and did not share it with his wife for a long time, instead resorting to alcohol consumption to cope. Sam similarly keeps his experience to himself and only speaks about it to his wife when, in a vulnerable state and within a supportive environment, he is able to engage in a liberating speech. Collective awareness among peers, thereby reducing stigmatization, is also a favorable factor in care (30), particularly when it occurs early in the posttraumatic period (33). In the same vein, this article aims to raise collective awareness of this disorder, thereby enhancing the probability of a favorable outcome when addressed early and comprehensively.

## 7 Conclusion

*Brothers* is an American film with interesting direction and strong acting, presenting a family drama in which episodes of war are intertwined with romantic storylines that raise questions about family, society, and American foreign policy. However, a sense of incompleteness and unresolved issues that appear to have been set aside—such as the internal experiences of the two girls or the abrupt end of the flirtation between Grace and Tommy—hang over the film. In light of the analysis presented in this article, multiple symptomatic criteria support the diagnosis of posttraumatic stress disorder, with the desire to return to combat standing out as the main element diverging from theoretical references. Another criticism is that the psychopathological movements and psychophysiological mechanisms are rendered in a correct but rather superficial way and viewed externally, without sufficient depth or internal development; a more detailed, nuanced, and explicit exploration would have facilitated a deeper apprehension of what torments the protagonist so profoundly. On another level, the family arrangements associated with the protagonist’s departure from and return to the closed family system are represented in a fine and nuanced way, with a newly found balance being overturned until the next crisis, when the trauma brought back from overseas resurfaces before finally being expressed verbally in a context of care. Moreover, this stereotypical representation conveys a stigmatizing image of patients suffering from PTSD, and the audience may come to believe, through exaggerated generalizations, that all patients with the disorder are unstable, violent, and dangerous. However, the film has the advantage of highlighting the problem of PTSD among veterans while raising the issue of psychiatric and psychosocial care for soldiers, combatants, and their families. In fact, the movie crystallizes the growing awareness of PTSD and the rising suicide rates among returning veterans. Treatment and support appear inadequate and poorly adjusted to their needs. *Brothers* raised awareness of postwar PTSD within the political landscape of its time by reflecting prevailing social concerns. In doing so, the film stressed the need for early screening, timely intervention, and access to mental health and addiction treatment for veterans and their families. It underscored the importance of addressing their health properly, holistically, and comprehensively.

## Data Availability

The original contributions presented in the study are included in the article/supplementary material. Further inquiries can be directed to the corresponding author.

## References

[1] Das S Doval N Mohammed S Dua N Chatterjee SS . Psychiatry and cinema: What can we learn from the magical screen? Shanghai Arch Psychiatry. (2017) 29:310–3. 29276355 10.11919/j.issn.1002-0829.217014 PMC5738520

[2] Arenas-Rojas AM . La relación entre el cine y la psiquiatría a través del tiempo y su rol en la educación médica actual. Rev Med Y Cine. (2021) 17:41–7. doi: 10.14201/rmc20211714147

[3] Damjanović A Vuković O Jovanović AA Jašović-Gašić M . Psychiatry and movies. In: Psychiatria danubina, vol. 21. (2009). p. 230–5. Available online at: https://hrcak.srce.hr/39897 (Accessed May 3, 2026). 19556954

[4] Delourmel C . From the function of the father to the paternal principle. Rev Française Psychoanalyse. (2013) 77:1283–353.

[5] De Neuter P . Transgenerational transmission. Clin Psychol Papers. (2014) 43:43–58. doi: 10.1521/ijgp.2008.58.3.389 18573029

[6] Eiguer A . Psychic and trans-generational transmission. Psy Field. (2011) 60:13–25.

[7] Nijenhuis ERS van der Hart O . Dissociation in trauma: A new definition and comparison with previous formulations. J Trauma Dissociation. (2011) 12:416–45. doi: 10.1080/15299732.2011.570592 21667387

[8] Flory JD Yehuda R . Comorbidity between post-traumatic stress disorder and major depressive disorder: Alternative explanations and treatment considerations. Dialogues Clin Neurosci. (2015) 17:141–50. doi: 10.31887/DCNS.2015.17.2/jflory 26246789 PMC4518698

[9] Fung HW Chien WT Lam SKK Ross CA . The relationship between dissociation and complex post-traumatic stress disorder: A scoping review. Trauma Violence Abuse. (2023) 24:2966–82. doi: 10.1177/15248380221120835 36062904

[10] Friedman MJ . Acknowledging the psychiatric cost of war. N Engl J Med. (2004) 351:75–7. doi: 10.1056/NEJMe048129 15229311

[11] Hoge CW Castro CA Messer SC McGurk D Cotting DI Koffman RL . Combat duty in Iraq and Afghanistan, mental health problems, and barriers to care. N Engl J Med. (2004) 351:13–22. doi: 10.1056/NEJMoa040603 15229303

[12] Harrison JE Weber S Jakob R Chute CG Reed GM Khoury B . International statistical classification of diseases and related health problems. 11th ed. Geneva, Switzerland: World Health Organization (2021).

[13] Javidi H Yadollahie M . Post-traumatic stress disorder. Int J Occup Environ Med. (2012) 3:2–9. 23022845

[14] Jordan AH Eisen E Bolton E Nash WP Litz BT . Distinguishing war-related PTSD resulting from perpetration- and betrayal-based morally injurious events. Psychol Trauma. (2017) 9:627–34. doi: 10.1037/tra0000249 28068142

[15] Juignet P . Paternal function and psychic maturity. In: Philosophy, science, and society. Schiltigheim, France: Libre Acces Editions (2015) (Accessed May 1, 2026).

[16] Júnior JMSS Oliveira JWC de Alencar RN Farias ATL Maia MM Peixoto GC . Cinema as a tool for teaching psychiatry: A systematic review. J Adv Med Med Res. (2025) 37:181–99. doi: 10.9734/jammr/2025/v37i85915 41757302

[17] Kilciksiz CM . Visual media: An aperture into the past and future of psychiatry. Am J Psychiatry. (2023) 19:2–23. doi: 10.1176/appi.ajp-rj.2023.190104 8723190

[18] Byrne P . Why psychiatrists should watch films (or what has cinema ever done for psychiatry)? Adv Psychiatr Treat. (2009) 15:286–96. doi: 10.1192/apt.bp.107.005306

[19] Ciccone A . Unconscious psychic transmission. In: Dunod (2012). p. 43.

[20] D’Agostini M Moltrasio C Feruglio S Crescentini C Paolone AR Fabbro F . Movies as innovative tools for teaching psychiatry: A new model for cinemedicine Vol. 65. Milan, Italy: Minerva Psychiatry (2024).

[21] Stein DJ Koenen KC Friedman MJ Hill E McLaughlin KA Petukhova M . Dissociation in posttraumatic stress disorder: Evidence from the world mental health surveys. Biol Psychiatry. (2013) 73:302–12. doi: 10.1016/j.biopsych.2012.08.022 23059051 PMC3589990

[22] Kimble M Boxwala M Bean W Maletsky K Halper J Spollen K . The impact of hypervigilance: Evidence for a forward feedback loop. J Anxiety Disord. (2014) 28:241–5. doi: 10.1016/j.janxdis.2013.12.006 24507631 PMC4211931

[23] Van Voorhees EE Dennis PA Elbogen EB Fuemmeler B Neal LC Calhoun PS . Characterizing anger-related affect in individuals with posttraumatic stress disorder using ecological momentary assessment. Psychiatry Res. (2018) 261:274–80. doi: 10.1016/j.psychres.2017.12.080 29329048 PMC6341481

[24] Sheridan J . Brothers. Universal Studios, California, USA: DreamWorks Pictures (2009).

[25] Scott AO . A war abroad ignites a battle at home. In: The new york times (2009). Available online at: https://www.nytimes.com/2009/12/04/movies/04brothers.html (Accessed May 3, 2026).

[26] Lester P Peterson K Reeves J Knauss L Glover D Mogil C . The long war and parental combat deployment: Effects on military children and at-home spouses. J Am Acad Child Adolesc Psychiatry. (2010) 49:310–20. doi: 10.1016/j.jaac.2010.01.003 20410724 PMC2875082

[27] Committee on the Assessment of the Readjustment Needs of Military Personnel, Veterans, and Their Families Board on the Health of Select Populations Institute of Medicine . Returning home from Iraq and Afghanistan: assessment of readjustment needs of veterans, service members, and their families. Washington (DC: National Academies Press (US (2013). 24901192

[28] Calzada Ribalta G Rothen S Machado A Penzenstadler L Thorens G Zullino D . The use of mainstream movies in psychiatric training. Swiss Arch Neurology Psychiatry Psychother. (2023) 174:110–3.

[29] Butler LD . The dissociations of everyday life. J Trauma Dissociation. (2004) 5:1–11. doi: 10.1300/J229v05n02_01 23861482

[30] Mittal D Drummond KL Blevins D Curran G Corrigan P Sullivan G . Stigma associated with PTSD: Perceptions of treatment seeking combat veterans. Psychiatr Rehabil J. (2013) 36:86–92. doi: 10.1037/h0094976 23750758

[31] Sreenivasan S Garrick T McGuire J Smee DE Dow D Woehl D . Critical concerns in Iraq/Afghanistan war veteran-forensic interface: Combat-related post deployment criminal violence. J Am Acad Psychiatry Law. (2013) 41:263–73. 23771940

[32] Tanielian T Jaycox LH . Invisible wounds of war: Psychological and cognitive injuries, their consequences, and services to assist recovery. RAND Corporation (2008). Available online at: https://www.jstor.org/stable/10.7249/mg720ccf (Accessed May 2, 2026).

[33] Wright BK Kelsall HL Sim MR Clarke DM Creamer MC . Support mechanisms and vulnerabilities in relation to PTSD in veterans of the Gulf War, Iraq War, and Afghanistan deployments: A systematic review. J Trauma Stress. (2013) 26:310–8. doi: 10.1002/jts.21809 23670878

